# Heredity in Consumption

**Published:** 1895-01-26

**Authors:** 


### Heredity in Consumption.

How far consumption is an hereditary disease is an interesting question, important not only to medical men but to all those who have a tradition of consumption in their family.

To marry or not to marry, to suckle babies or to use the bottle, to enter on the work of life or to coddle and leave the struggle to the strong; all these are questions the answer to which largely depends on whether or no an hereditary taint is to be reckoned with as an important factor in the production of consumption.

It may be, and, in fact, has been, said that this is a matter which typically lends itself to statistical inquiry. But there are many difficulties in the way; not only must a definition be given of what one means by consumption, but we must make sure that other people mean the same, for the rough and ready statements people make as to the ailments of their relatives are open to many doubts. Nor must it be forgotten that hereditary influence is by no means excluded by seemingly healthy parentage. Healthy parents may be but the survivors of very weakly families, and seeing the large proportion of the people carried off by this disease, even the strongest families must often intermingle with those in which consumption has occurred. On the other hand, it is asserted that the most complete statistical evidence that phthisis runs in families is no proof that it is hereditary, for, according to the theory of infection, it is clear that the offspring of consumptive families, brought up in the midst of possible infection, run more risk of catching the disease than the generality of people.

Putting general statistics on one side, there are few medical men who could not tell of families in which the presence of consumption has been common in an extraordinary degree. Again, as was lately pointed out by Dr. Pollock at the Medical and Chirurgical Society, in the discussion on a paper by Dr. Ed. Squire, not only do certain families suffer more than others, but certain forms of the disease appear to prevail in some families generation after generation. This brings one at once to the point as to the nature of the thing transmitted, in regard to which there is very strong evidence that although the transmission of the disease itself is a possibility, and has been proved to occur in a few instances in the case of calves yet, even giving the widest latitude to the possibility of prolonged latency, it is by no means the ordinary way in which hereditary influence acts, and that, so far as this does act, it is in the transmission of a tendency to, or a lessened resistance to the development of, the micro-organism of tuberculosis. The relation which the tendency bears to the fully-developed disease is well exemplified by a consideration of what happens to certain monkeys, which when imported to England from their primeval forests commonly die of consumption. That they have an inborn tendency to succumb to phthisis appears certain, notwithstanding that none of their ancestors ever died of it, and we cannot but doubt that, like these apes, members of certain families more than others tend to die of consumption if it comes in their way. Dr. Squire's contention at the meeting above referred to, that the children of consumptives do this just as they take other diseases, because they are of a weakly constitution may be true in a sense, but it in no way involves the opposite proposition, viz., that strong people are safe from phthisis by virtue of their strength. Dr. Heron's remarks concerning the great mortality among our Foot Guards years ago, and among the nursing orders in Prussia, two classes of people who had been carefully examined and certified to be in good health before they took up their work, were sufficient to show that the tendency to phthisis has but little necessary relation to what may be called general debility. That the tendency to take the tuberculous infection is something strange, curious, and ill understood, like that to take scarlet fever badly, a family tendency that everybody admits, may then well be allowed; but that it is the main factor in the production of the disease must be denied. Tuberculosis is an infective disease, but practically it is only infectious where certain causes co-operate with the bacillus or contagious particle, such as a confined occupation, absence of fresh air, want of exercise, inefficient diet, life in a dirty, dusty house, or in dusty offices or work-rooms, the dust in which has become charged with the bacilli of tuberculosis, and lastly, a special susceptibility to the disease, either innate or left by some other malady; these are the causes of consumption, and of these the hereditary tendency is but one, and we can well agree with Dr. Heron's remark at the Medical and Chirurgical Society that Dr. Squire's paper will help certain unfortunate families to get rid of the terrible dread which haunts them in consequence of having been brought up to believe that since their father or mother, or grandfather or grandmother died of tuberculosis, they themselves, therefore, run a special risk of the same disease. They certainly should avoid occupations and modes of life known to predispose to it, but otherwise they need not fear.

